# An Associative Memory Approach to Healthcare Monitoring and Decision Making

**DOI:** 10.3390/s18082690

**Published:** 2018-08-16

**Authors:** Mario Aldape-Pérez, Antonio Alarcón-Paredes, Cornelio Yáñez-Márquez, Itzamá López-Yáñez, Oscar Camacho-Nieto

**Affiliations:** 1 Instituto Politécnico Nacional, Computational Intelligence Laboratory at CIDETEC, Ciudad de Mexico 07700, Mexico; ilopezy@ipn.mx (I.L.-Y.); ocamacho@ipn.mx (O.C.-N.); 2 Universidad Autónoma de Guerrero, Engineering Department, Guerrero 39079, Mexico; aalarcon@uagro.mx; 3 Instituto Politécnico Nacional, Computational Intelligence Laboratory at CIC, Ciudad de Mexico 07738, Mexico; cyanez@cic.ipn.mx

**Keywords:** associative memories, decision support systems, e-Health, Internet of Things, pattern classification

## Abstract

The rapid proliferation of connectivity, availability of ubiquitous computing, miniaturization of sensors and communication technology, have changed healthcare in all its areas, creating the well-known healthcare paradigm of e-Health. In this paper, an embedded system capable of monitoring, learning and classifying biometric signals is presented. The machine learning model is based on associative memories to predict the presence or absence of coronary artery disease in patients. Classification accuracy, sensitivity and specificity results show that the performance of our proposal exceeds the performance achieved by each of the fifty widely known algorithms against which it was compared.

## 1. Introduction

In a large number of countries, healthcare systems and services have become an essential human right; consequently, individuals’ access to the public health systems has become an indicator of the well-being and the development of nations [1,2]. Public health systems were designed to attend and provide health services to specific individuals. However, the constant growth of population and the increasing costs of health services imply that the public health system will face new challenges [3]. Some important aspects to consider are to develop and evaluate innovative approaches for improving the quality of healthcare using sensors applications in medical monitoring. Two decades ago, technology researchers took an important role towards improving the medical care of patients through the evolution of the concept of a network of smart devices, which would be known as Wireless Sensor Networks (WSNs) [4]. In the same decade, the concept of moving small amounts of data to a large set of nodes evolved to what today is known as the Internet of Things (IoT) [5,6]. The IoT paradigm represents one of the most disruptive technologies, enabling ubiquitous computing scenarios for medical monitoring, and decision making [7,8]; creating the well-known healthcare paradigm of e-Health [9]. This paradigm arises as a result of the combination of emerging technologies, such as IoT, ubiquitous computing, WSNs, high-speed communications infrastructure, and the social need for more effective health services with better accessibility and availability [10]. The e-Health paradigm has changed the traditional way in which healthcare services are provided. With this paradigm the user of healthcare services does not need to move to medical facilities to carry out a routine follow-up [11,12]; on the contrary, medical monitoring can be carried out from where the patient is located [13,14]. In addition, all acquired data can be transmitted, processed and stored for data mining and decision making by medical specialists [15,16]. Nowadays, it is increasingly common to use applications based on artificial intelligence techniques to support medical specialists in decision making. For more than a decade, statistical techniques [17], expert systems [18], neural networks [19], decision trees [20] and associative memories [21,22] have been widely used for pattern recognition, feature selection, data mining and decision making in the medical field [23,24,25].

In this paper, an embedded system capable of monitoring, learning and classifying biometric signals is presented. The machine learning model is based on associative memories to predict the presence or absence of coronary artery disease in patients. Classification accuracy, sensitivity and specificity results show that the performance of our proposal exceeds the performance achieved by each of the fifty widely known algorithms against which it was compared.

The paper is organized as follows. Section 2 presents previous works related to machine learning based systems that have been applied to predict the presence or absence of coronary artery disease in patients. In Section 3, a succinct description of associative memories fundamentals is presented. In Section 4, an improvement to Delta Associative Memory original model, called IDAM, is proposed. Section 5 presents the three main performance indicators of a binary classification test. The experimental phase is described in Section 6. In Section 7, sensitivity, specificity and classification accuracy results achieved by each of the compared algorithms in two datasets related to coronary artery disease diagnosis, are presented. Finally, our proposal’s advantages, as well as some conclusions are discussed in Section 8.

## 2. Previous Works

For more than a decade, machine learning based systems have been tested in patients with cardiac disease to predict outcome, or in the general population to detect cardiac diseases. In 2008, Kahramanli and Allahverdi [26] proposed a hybrid neural system for heart diseases that includes artificial neural network (ANN) and fuzzy neural network (FNN); the dataset was obtained from the University of California at Irvine (UCI) machine learning repository [27]. In 2009, Polat and Güneş [28] proposed a feature selection method on classification of medical datasets called Kernel F-score feature selection (KFFS); experimental results showed that KFSS achieved better results compared to F-score feature selection (FFS). In 2011, McSherry [29] proposed an approach to conversational case-based reasoning (CCBR) in medical classification and diagnosis that aims to increase transparency while also providing high levels of accuracy and efficiency; two datasets from the UCI machine learning repository were used in the experimental phase. In 2012, Aldape-Pérez et al. [23] proposed an associative memory approach to medical decision support systems. This work focuses on the use of classical associative memories for medical patterns classification. This approach incorporates a learning reinforcement stage, which increases the classification performance of classical models of associative memories. The performance was validated on medical datasets collected from the UCI machine learning repository. In 2012, Anooj [30] proposed a weighted fuzzy rule-based clinical decision support system (CDSS) for the diagnosis of coronary artery disease; the experimentation was carried out on the proposed system using the datasets obtained from the UCI machine learning repository and the performance of the system was compared with a neural network-based system utilizing accuracy, sensitivity and specificity. In 2013, Nahar et al. [31] proposed a computational intelligence approach for association rule mining to investigate the sick and healthy factors which contribute to coronary artery disease for males and females; the dataset that was used in the experimental phase was the UCI Cleveland dataset. In 2014, Biswas et al. [32] proposed a method to extract symbolic weights from a trained neural network by observing the whole trained neural network as an AND/OR graph and then finding a solution for each node that becomes the weight of a corresponding node. The performance was validated on coronary artery disease dataset collected from the UCI machine learning repository. In 2015, Aldape-Pérez et al. [24] proposed a collaborative learning approach based on associative models to pattern classification in medical datasets. In this work, Delta Associative Memory was presented. The operation of this model is based on the differences that exist between patterns of different classes and a dynamic threshold that is calculated for each unknown pattern to be classified. The experimental results were competitive, when compared against algorithms in the current literature. In 2015, Nguyen et al. [33] proposed an integration of fuzzy standard additive model (SAM) with genetic algorithm (GA), called GSAM, to deal with uncertainty. The proposed method was evaluated using Cleveland coronary artery disease dataset from the UCI machine learning repository. In 2016, Leema et al. [34] proposed a Computer-Aided Diagnostic (CAD) system that uses an Artificial Neural Network (ANN) trained by Differential Evolution (DE), Particle Swarm Optimization (PSO) and gradient descent based backpropagation (BP) for classifying clinical datasets, obtained from the UCI machine learning repository. In 2016, Nahato et al. [35] proposed a classifier that combines the fuzzy sets and extreme learning machine (FELM) for clinical datasets. The three major subsystems in the FELM framework are preprocessing subsystem, fuzzification subsystem and classification subsystem. Missing value imputation and outlier elimination are handled by the preprocessing subsystem. Cleveland coronary artery disease dataset from the UCI machine learning repository was used for experimentation. In 2017, Ramírez-Rubio et al. [25] proposed an associative model called Normalized Difference Associative Memory. This associative model overcome the limitations of the original Alpha-Beta Associative Memories [36]. In 2017, Shah et al. [37] proposed a methodology which uses the results of medical tests as input, extracts a reduced dimensional feature subset and provides diagnosis of coronary artery disease. The proposed methodology extracts high impact features in a new projection by using Probabilistic Principal Component Analysis (PPCA). The feature subset with the reduced dimension is provided to radial basis function (RBF) kernel based Support Vector Machines (SVM). Methodology performance was evaluated through accuracy, specificity and sensitivity over the three datasets of the UCI machine learning repository.

## 3. Associative Memories

The first models of Associative Memories arise with the scientific findings of Steinbuch in the 1960s [38,39,40], which over time would be known as Learning Matrices. In any learning matrix, there are two phases that determine the performance of each model, namely learning phase and classification phase. Learning matrices are structures formed by rows and columns whose intersection points are formed by connecting elements [41]. The characteristics of an object are presented during the learning phase to the columns as binary signals via a suitable transducer. Simultaneously, a meaning of an object associated with this set of characteristics is applied in the form of a signal to one of the rows. Therefore, so-called conditioned connections are effected in the connective elements of the row selected by the meaning [42]. Generalizing, a conditioned connection is a functional connection between a row and a column. In this way, during the learning phase each input vector $x^{\mu }\in A^{n},$ A={0,1} (characteristics of an object) forms an association with its corresponding output vector $y^{\mu }\in A^{m}$ (meaning of an object associated with this set of characteristics), so for each γ integer and positive, the corresponding association is denoted as: $\left(x^{\gamma },y^{\gamma }\right)$. Thus, an associative memory M is generated from an a priori finite set of known associations, called the fundamental set of associations. If μ is an integer and positive value, the fundamental set is represented as: $\left\{\left(x^{\mu },y^{\mu }\right)|\mu =1,2,\ldots ,p\right\}$ with *p* as the cardinality of the set. A distorted version of a pattern $x^{\gamma }$ to be recalled is denoted as ${\tilde{x}}^{\gamma }$. An unknown input pattern to be recalled is denoted as $x^{\omega }$. If when an unknown input pattern $x^{\omega }$ is fed to an associative memory M, it happens that the output corresponds exactly to the associated pattern $y^{\omega }$, it is said that recalling is correct.

Associative memories have been widely used to perform pattern recognition tasks effectively, however, they present a limitation known as *cross-talk*. The influence of *cross-talk* causes the associative memory to become saturated and consequently the classification performance is negatively affected.

## 4. Our Proposal

Negative effects of *cross-talk* are due to an order relation between patterns that constitute the fundamental set $\left\{\left(x^{\mu },y^{\mu }\right)|\mu =1,2,\ldots ,p\right\}$ with *p* as the cardinality of the set [36]. To improve classification performance of Delta Associative Memory [24], as well as to eliminate the negative effects of *cross-talk*, an improvement to Delta Associative Memory model is proposed, called Improved Delta Associative Memory (IDAM). This modification consists of adding a data preprocessing stage before the Delta Associative Memory learning phase. This additional stage is based on information quality estimation concepts that were proposed by Aldape-Pérez et al. [23] to reinforce learning in an associative memory. In the present paper, those concepts are used but with very different purposes, namely: to obtain a transformed fundamental set of patterns. The details of Delta Associative Memory model can be reviewed in Reference [24]. It should be noted that both the learning phase and the classification phase of Delta Associative Memory model remained unchanged. For clarity purposes, in the present paper, the same symbology is used.

### Preprocessing Phase

Data preprocessing phase is applied before Delta Associative Memory learning phase. This phase transforms the values of the input patterns of the fundamental set. This transformation of the input patterns is a data translation process that does not affect its representation or its statistical distribution. Furthermore, negative effects of *cross-talk* are eliminated; consequently, classification performance is improved. The proposed algorithm is as follows (Algorithm 1):

**Algorithm 1:** Preprocessing phase

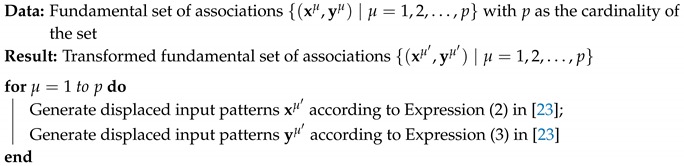



## 5. Performance Evaluation Methods

There are three main performance indicators of a binary classification test: sensitivity, specificity and classification accuracy. These indicators are computed from the confusion matrix values. Sensitivity and specificity are used for assessing the results of diagnostic and screening tests [43]. Sensitivity or True Positive Rate (TPR) represents the proportion of truly diseased persons in a screened population who are identified as being diseased by the test. Sensitivity is a measure of the probability of correctly diagnosing a condition. Specificity or True Negative Rate (TNR) is the proportion of truly healthy persons who are identified as so by the screening test. Classification accuracy of any algorithm can be estimated by taking into account the overall number of test patterns that are correctly classified.

## 6. Experimental Phase

The experimental phase of this paper is divided into two parts. In the first part, the coronary artery disease dataset, taken from the University of California at Irvine (UCI) machine learning repository [27], was used to evaluate the performance of the proposed model. The results of sensitivity, specificity and classification were compared against the performance achieved by fifty widely known algorithms, available in WEKA 3: Data Mining Software in Java [44]. The purpose of this stage is to evaluate the performance of the proposed algorithm using data that are still widely used by the scientific community. Cross-validation (CV) was used as a technique to assess the generalizability of the proposed model to unknown patterns; specifically, *k*-fold cross-validation with k=10 was used.

**Figure 1:**
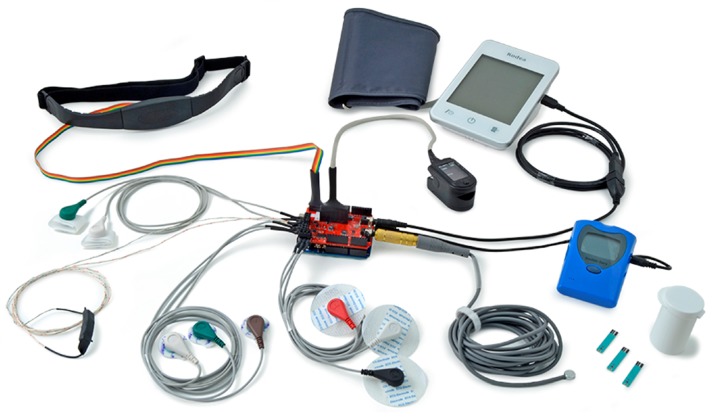
The platform allows us to monitor biometric signals by using different sensors (courtesy of Libelium).

In the second part, the Sensor Platform shown in Figure 1 was integrated to a computing device based on the single-board computer paradigm. In this device, the proposed associative memory model was implemented and a database of medical patterns was generated. Once the device was trained, we proceeded with the tests of unknown patterns, generating classification performance results. To ensure the experimental results are reliable and valid on unknown patterns, all experiments were carried out following Kohavi and John recommendations [45]. Classification performance, sensitivity and specificity of the proposed model was compared against fifty widely known models, available in WEKA 3: Data Mining Software in Java [44].

### 6.1. Heart Disease Dataset

This dataset comes from the Cleveland Clinic Foundation and was supplied by Robert Detrano, M.D., Ph.D. of the V.A. Medical Center, Long Beach, CA, USA. The purpose of the dataset is to predict the presence or absence of coronary artery disease given the results of various medical tests carried out on a patient. This dataset consists of 270 instances belonging to two different classes: presence and absence (of coronary artery disease). Each instance consists of 14 attributes, including the class attribute. This dataset and more information about the attributes are available at the University of California at Irvine (UCI) machine learning repository [27].

### 6.2. e-Health Sensor Platform Dataset

This dataset was created using the e-Health Sensor Platform, shown in Figure 1. It was built with the approval of the participants of the Research Projects 20130307 and 20140461, registered in the National Polytechnic Institute of Mexico (IPN). The objectives and goals of the projects were explained to each of the participants. Personally identifiable information was removed so that the dataset is anonymized. The purpose of the dataset is to predict the presence or absence of coronary artery disease given the results of various medical tests carried out on a patient. This dataset consists of 135 instances belonging to two different classes: presence and absence (of coronary artery disease). Each instance consists of seven attributes, including the class attribute. Attribute Information is as follows:

1. age
2. sex
3. maximum heart rate achieved
4. resting electrocardiographic results (values 0, 1, 2)
5. fasting blood sugar >120 mg/dL
6. resting blood pressure
7. class attribute: presence or absence (of coronary artery disease)

## 7. Results and Discussion

Classification performance of our proposal was compared against fifty widely known classification models. Table 1 and Table 2 show classification performance, sensitivity and specificity achieved by the twenty best-performing algorithms of the fifty widely known algorithms, available in WEKA 3: Data Mining Software in Java [44].

According to the type of learning scheme, each of these can be grouped into one of the following types of classifiers: Functions based classifiers, Meta classifiers, Rules based classifiers, Bayesian classifiers and Decision Trees classifiers.

The twenty best-performing algorithms are as follows:

- Four functions based classifiers (Logistic [46], RBFNetwork [47], SimpleLogistic [48] and SMO [49]).
- Seven meta classifiers (AdaBoostM1 [50], Bagging [51], Dagging [52], MultiClassClassifier [53,54], RandomCommittee [53,54], RandomSubSpace [55], RotationForest [56]).
- Two rules based classifiers (DecisionTable [57] and DTNB [58]).
- Four algorithms based on the Bayesian approach (BayesNet [59], NaiveBayes [60], NaiveBayesSimple [61] and NaiveBayesUpdateable [60]).
- Three decision trees classifiers (FT [62], LMT [62], and RandomForest [63]).

Table 3 and Table 4 show classification accuracy achieved by the five best-performing algorithms of the fifty widely known classification models.

As shown in Table 1, the algorithm that best identifies sick patients, using coronary artery disease dataset, is RandomForest with a Sensitivity value of 89.30. The model that best identifies healthy patients is Improved Delta Associative Memory which achieved a Specificity value of 80.83. As shown in Table 3, RBFNetwork algorithm and Improved Delta Associative Memory achieved the highest classification accuracy.

Performance achieved by Improved Delta Associative Memory is very competitive, as can be seen in Table 2. There are two algorithms that best identify healthy patients, using e-Health Sensor Platform Dataset, SimpleLogistic and Dagging with a Specificity value of 98.00. The model that best identifies sick patients is Improved Delta Associative Memory which achieved a Sensitivity value of 98.33. As shown in Table 4, Improved Delta Associative Memory algorithm achieved the highest classification accuracy.

As shown in Table 1 and Table 2, there is no particular method that surpasses all other algorithms. Wolpert and Macready [64] proved that what an algorithm gains in performance on one class of problems is necessarily offset by its performance on the remaining problems.

As shown in Table 3 and Table 4, Improved Delta Associative Memory has a competitive performance compared against the performance achieved by the fifty widely known algorithms, available in WEKA 3: Data Mining Software in Java [44]. It is worth noting that Improved Delta Associative Memory achieved the best performance using the e-Health Sensor Platform Dataset. Similarly, it should be noted that Improved Delta Associative Memory achieved the best classification accuracy averaged over the two datasets.

It is necessary to highlight that, using e-Health Sensor Platform Dataset, Improved Delta Associative Memory model achieved the best classification performance as well as the highest capacity to identify sick patients.

As can be seen in Table 4, in the two most important values for medical diagnosis and decision making, Improved Delta Associative Memory model delivered the best performance.

## 8. Conclusions

The proposed model, called Improved Delta Associative Memory, showed competitive performance compared against the performance achieved by the fifty widely known algorithms, available in WEKA 3: Data Mining Software in Java [44].

It should be noted that Improved Delta Associative Memory achieved the best classification accuracy averaged over all datasets. Likewise, in the two most important values for medical diagnosis and decision making, Improved Delta Associative Memory model delivered the best performance.

Classification performance of Improved Delta Associative Memory demonstrates associative memories potential to develop applications based on artificial intelligence techniques to support medical specialists in healthcare monitoring and decision making.

The results presented in this paper demonstrate associative memories potential to predict the presence or absence of coronary artery disease for pattern classification systems.

## Figures and Tables

**Table 1:** Classification accuracy using Heart Disease Dataset. Algorithms are presented in alphabetical order.

No	Algorithm	Sensitivity	Specificity	Accuracy
1.	AdaBoostM1	85.30	78.30	82.22
2.	Bagging	87.30	79.20	83.70
3.	BayesNet	86.00	77.50	82.22
4.	Dagging	88.00	75.00	82.22
5.	DecisionTable	87.30	78.30	83.33
6.	DTNB	85.30	79.20	82.59
7.	FT	86.00	77.50	82.22
8.	LMT	86.00	77.50	82.22
9.	Logistic	87.30	79.20	83.70
10.	MultiClassClassifier	87.30	79.20	83.70
11.	NaiveBayes	87.30	78.30	83.33
12.	NaiveBayesSimple	86.70	78.30	82.96
13.	NveBayesUpdateable	87.30	78.30	83.33
14.	RandomCommittee	86.70	76.70	82.22
15.	RandomForest	89.30	76.70	83.70
16.	RandomSubSpace	86.70	76.70	82.22
17.	RBFNetwork	86.70	80.83	84.07
18.	RotationForest	86.70	77.50	82.59
19.	SimpleLogistic	86.00	77.50	82.22
20.	SMO	86.70	79.20	83.33
21.	IDAM (our proposal)	86.70	80.83	84.07

**Table 2:** Classification accuracy using e-Health Sensor Platform Dataset. Algorithms are presented in alphabetical order.

No	Algorithm	Sensitivity	Specificity	Accuracy
1.	AdaBoostM1	94.10	96.40	95.60
2.	Bagging	95.40	96.60	96.19
3.	BayesNet	97.90	96.80	97.21
4.	Dagging	94.60	98.00	96.77
5.	DecisionTable	93.70	96.80	95.75
6.	DTNB	98.30	97.10	97.51
7.	FT	97.50	96.60	96.92
8.	LMT	94.10	97.70	96.48
9.	Logistic	94.60	97.70	96.63
10.	MultiClassClassifier	94.60	97.70	96.63
11.	NaiveBayes	97.10	95.70	96.19
12.	NaiveBayesSimple	97.90	95.50	96.33
13.	NveBayesUpdateable	97.10	95.70	96.19
14.	RandomCommittee	95.40	97.10	96.48
15.	RandomForest	97.50	96.80	97.07
16.	RandomSubSpace	95.00	96.20	95.54
17.	RBFNetwork	95.80	95.90	95.90
18.	RotationForest	97.90	96.80	97.21
19.	SimpleLogistic	94.10	98.00	96.63
20.	SMO	95.80	97.50	96.92
21.	IDAM (our proposal)	98.33	97.51	97.80

**Table 3:** Classification accuracy of the five best-performing algorithms using Heart Disease Dataset.

No	Algorithm	Sensitivity	Specificity	Accuracy
1.	Bagging	87.30	79.20	83.70
2.	Logistic	87.30	79.20	83.70
3.	RandomForest	89.30	76.70	83.70
4.	RBFNetwork	86.70	80.83	84.07
5.	IDAM (our proposal)	86.70	80.83	84.07

**Table 4:** Classification accuracy of the five best-performing algorithms using e-Health Sensor Platform Dataset.

No	Algorithm	Sensitivity	Specificity	Accuracy
1.	BayesNet	97.90	96.80	97.21
2.	DTNB	98.30	97.10	97.51
3.	RotationForest	97.90	96.80	97.21
4.	SimpleLogistic	94.10	98.00	96.63
5.	IDAM (our proposal)	98.33	97.51	97.80
